# Use of Insulin and Mortality from Breast Cancer among Taiwanese Women with Diabetes

**DOI:** 10.1155/2015/678756

**Published:** 2015-06-11

**Authors:** Chin-Hsiao Tseng

**Affiliations:** ^1^Department of Internal Medicine, National Taiwan University College of Medicine, Taipei, Taiwan; ^2^Division of Endocrinology and Metabolism, Department of Internal Medicine, National Taiwan University Hospital, Taipei, Taiwan; ^3^Division of Environmental Health and Occupational Medicine, National Health Research Institutes, Zhunan, Taiwan

## Abstract

*Background*. To evaluate whether insulin use was predictive for mortality from breast cancer in Taiwanese women with diabetes mellitus. *Methods*. A total of 48,880 diabetic women were followed up to determine the mortality from breast cancer during 1995–2006. Cox models were used, considering the following independent variables: age, sex, diabetes type, diabetes duration, body mass index, smoking, insulin use, and area of residence. Insulin use was also considered for its duration of use at cutoffs of 3 years and 5 years. *Results*. Age was a significant predictor in all analyses. The multivariable-adjusted hazard ratio (95% confidence interval, *P* value) for insulin use without considering the duration of use was not statistically significant (1.339 [0.782–2.293, *P* = 0.2878]). Compared with nonusers, insulin users showed the following adjusted hazard ratios for insulin use <3 years, ≥3 years, <5 years, and ≥5 years: 0.567 (0.179–1.791, *P* = 0.3333), 2.006 (1.102–3.653, *P* = 0.0228), 1.045 (0.505–2.162, *P* = 0.9048), and 1.899 (0.934–3.860, *P* = 0.0763). *Conclusions*. Insulin use (mainly human insulin) for ≥3 years may be associated with a higher risk of breast cancer mortality.

## 1. Introduction

Patients with diabetes may suffer from a higher risk of cancer involving the liver, pancreas, endometrium, colorectum, bladder, and breasts [1]. The use of antidiabetic drugs may also be associated with a higher risk of various types of cancer [2–6]. Recently, we reported that female patients with diabetes in Taiwan had a significantly higher risk, ranging from 1.37-fold to 2.43-fold in different age groups, of mortality from breast cancer, compared with the general population [7]. Epidemiological studies suggest that the link between diabetes and breast cancer may be attributed to the aberration in glucose metabolism and the presence of insulin resistance [8]. However, detection bias may also explain part of the increased risk of breast cancer in female patients with diabetes [9]. In addition, serum levels of insulin [10, 11], C-peptide [12], insulin-like growth factors, and insulin-like growth factor binding proteins [10] are predictive for breast cancer. The prognosis of breast cancer may also be affected by diabetes status [13] and fasting serum insulin level [14].

In addition to its metabolic effect, insulin has mitogenic properties and can regulate cell proliferation, differentiation, and apoptosis [1]. Studies also suggested that breast epithelial cells contain insulin receptors, which are upregulated in breast cancer cells [15], and insulin mitogenic pathway can be exaggerated in the presence of insulin resistance [16]. Therefore, an elevated serum level of insulin can theoretically cause breast cancer via the crosstalk between insulin and insulin receptor and/or insulin-like growth factor receptors, especially in the presence of insulin resistance [17]. However, whether the use of exogenous insulin (i.e., human insulin or insulin analogues) for treating hyperglycemia in patients with diabetes actually increases the risk of breast cancer is the subject of recent debate [18–20].

Because the prevalence of diabetes is increasing and insulin is used to treat millions of diabetic patients, it is clinically important to clarify the effects of insulin use on the risk for breast cancer. Therefore, this study evaluated whether the use of insulin in a nationally representative cohort of female patients with diabetes in Taiwan increased the risk of mortality due to breast cancer, over a 12-year period of followup.

## 2. Materials and Methods

### 2.1. Study Population

Since March 1995, a compulsory and universal National Health Insurance program was implemented in Taiwan, which encompassed >96% of the entire population of Taiwan. The establishment of a cohort of diabetic patients with a structured baseline questionnaire using the National Health Insurance for long-term followup by telephone interview was approved by an ethics committee of the Department of Health, Executive Yuan, Taiwan. The detailed processes for the establishment of the study cohort, the compilation of data, and the questionnaire interview were described previously [7]. In brief, from 1995 to 1998, a cohort of 256,036 patients with diabetes using the insurance was identified [7]. Half of the identified diabetic patients were randomly selected for the questionnaire interview (*n* = 128,572), and among them, 93,484 (72.7%) were successfully interviewed by well-trained interviewers. Verbal informed consent from the participants was given after the purpose of the research was explained to them, and they were informed that the study was conducted under the approval of the Department of Health. Written informed consent was not obtained because it was not a necessary requirement for telephone questionnaire interviews, according to local regulations, at the time when the study was approved and the interviews were conducted.

The interviewers submitted the questionnaires every week, and all returned questionnaires were checked for internal consistency by an assistant and then again by the investigator (C.-H. T.). The information obtained during the interview included demographic data, self-reported height and weight, ethnic background of both parents (respondents were asked to choose from 5 categories [Fukien, Hakka, Mainlander, Aborigine, or other]), history of lower-extremity amputation, initial symptoms or clinical events that led to the diagnosis of diabetes, current treatment of diabetes, history of smoking and use of betel nut, hypertension, family history of diabetes and hypertension, and area of residence.

**Figure 1:**
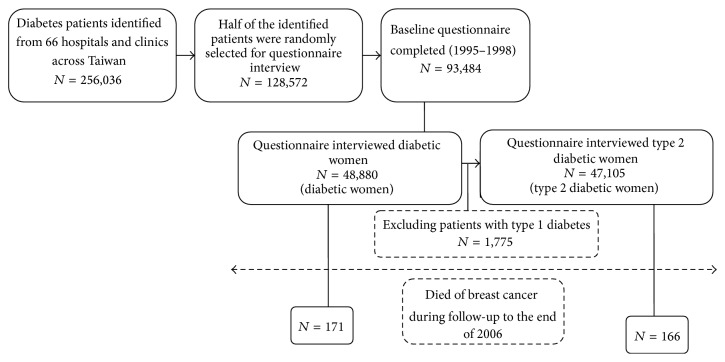
Flow chart showing the procedures for the followup of women with diabetes from a nationally representative cohort for breast cancer mortality.

Figure 1 shows a flow chart of the process for followup of the diabetic women from the nationally representative cohort for breast cancer mortality. Diagnosis of diabetes in Taiwan was made as per the definition of diabetes by the World Health Organization in clinical practice. Diabetic patients in the present study were defined as patients who had been clinically diagnosed as having diabetes and were being treated for the disease in a medical setting. The diagnosis of diabetes in the interviewed individuals was confirmed by questions in the questionnaire about the age of onset, the initial symptoms or clinical events that led to the diagnosis, and the current treatment of diabetes.

The following variables were abstracted for analysis in the present study: age, diabetes duration, diabetes type (diagnosis of type 1 diabetes mellitus was based on a history of diabetic ketoacidosis at diabetes onset or the need for insulin injection within 1 year after diabetes diagnosis), body mass index (calculated as body weight in kilograms divided by squared body height in meters, based on self-reported information), smoking, insulin use, and area or residence.

### 2.2. Ascertainment of Vital Status

In Taiwan, every resident has a unique identification number, and events such as birth, death, marriage, and migration should be registered in the household registration offices. The data included on the death certificates, including the unique identification number, date of birth, sex, and date and cause of death, have been computerized since 1971 and can be used for academic research. Since 1981, causes of death have been coded according to the ninth revision of the International Classification of Diseases in Taiwan. The code for death due to breast cancer is 174.

### 2.3. Risk Factors for Breast Cancer Mortality

All patients were followed until December 31, 2006. The dates and causes of death for those who died were obtained by matching with the national death certificate database. A total of 171 patients died of breast cancer, and among them, only 5 had type 1 diabetes mellitus (Figure 1).

The Cox proportional hazards model was used to evaluate the risk factors associated with breast cancer mortality in the whole cohort, in which breast cancer mortality was the dependent variable and the independent variables included age, diabetes type (type 2 or type 1), diabetes duration, body mass index, smoking (yes or no), insulin use (yes or no), and area of residence (urban or rural). The area of residence was defined as urban for the Metropolitan Taipei area (including Taipei city and Taipei county) and other administratively named cities across Taiwan and as rural for administratively named counties and offshore islands. Sensitivity analyses for the abovementioned Cox regression model were also created after excluding patients who died of breast cancer within 3 years, 5 years, and 7 years of diabetes onset, respectively, to minimize the possibility that diabetes may be caused by incipient breast cancer or may occur after the diagnosis of breast cancer.

To further examine whether the risk of breast cancer mortality might vary with the duration of insulin use, Cox models were also created by comparing insulin use for <3 years and ≥3 years to nonuse and that for <5 years and ≥5 years to nonuse, after adjustment for all covariates in the whole cohort (whole cohort analyses). Additional fully adjusted models were created for various causes of mortality as dependent variables to examine whether the association between duration of insulin use might differ for different causes of mortality. The following causes of mortality were evaluated: all-cause, all-noncancer, all-cancer, and all cancer other than breast cancer. For breast cancer mortality, additional models were created for estimating hazard ratios and their 95% confidence intervals for insulin use for ≥3 (or ≥5) years versus nonuse/use for <3 (or <5) years.

Because insulin use is highly dependent on the duration of diabetes, residual confounding effect from diabetes duration might not be completely excluded in the abovementioned analyses of the whole cohort. To completely exclude such a residual confounding effect, additional analyses were conducted in a subgroup of patients comprised of all cases of breast cancer mortality plus a group of randomly selected patients without breast cancer mortality, matched by diabetes duration, with a ratio of 1 : 5. These analyses estimated the adjusted hazard ratios and their 95% confidence intervals for insulin use for <3 (or <5) years and ≥3 (or ≥5) years as compared to nonuse and insulin use for ≥3 (or ≥5) years as compared to nonuse/use <3 (or <5) years.

Analyses were conducted using the SAS statistical software, version 9.3 (SAS Institute, Cary, NC). *P* < 0.05 was considered statistically significant.

## 3. Results

A total of 48,880 female patients were included in the present study (Figure 1).  Table 1 compares the baseline characteristics between the diabetic women who died (*n* = 171) and those who did not die (*n* = 48,709) of breast cancer. Patients who died of breast cancer had significantly older age, with a higher proportion aged ≥65 years (48.5% versus 39.9%), and a higher proportion of living in urban areas (55.0% versus 45.9%). In addition, in these women, mean diabetes duration was longer (8.5 years versus 7.5 years) but with borderline significance (*P* = 0.0706). The other variables, including use of insulin, diabetes type, body mass index, and smoking, did not differ significantly between the 2 groups. For those who died of breast cancer and those who did not, the median duration of followup was 3.7 and 7.4 years, respectively, and the median duration of insulin use in insulin users in the 2 groups was 6.0 and 4.0 years, respectively. The median follow-up duration for insulin users and nonusers in the cohort was 7.3 years and 7.5 years, respectively.

Table 2 shows the mutually adjusted hazard ratios and their 95% confidence intervals, with and without excluding patients who died of breast cancer within 3 years of diabetes onset. Age was consistently a significant predictor. Though not statistically significant, insulin use was associated with a higher risk of breast cancer mortality, with adjusted hazard ratio (95% confidence interval) of 1.339 (0.782–2.293) and 1.384 (0.806–2.375), respectively. Sensitivity analyses conducted after excluding patients who died of breast cancer within 5 years and 7 years of diabetes onset, separately, did not remarkably change the adjusted hazard ratios (data not shown).

Table 3 shows the multivariable-adjusted hazard ratios for various causes of mortality including breast cancer, all-cause, all-noncancer, all-cancer, and all cancer other than breast cancer with regard to 2 cutoffs of duration of insulin use at 3 years and 5 years, respectively, in the whole cohort analyses and in the subgroup analyses. The hazard ratios for breast cancer mortality for insulin use for ≥3 years were consistently and significantly higher than that in the comparison groups in all analyses. Patients who used insulin for ≥5 years also consistently had a higher risk of breast cancer mortality compared with the comparison groups, but with a borderline statistical significance (0.05 < *P* < 0.1). Insulin use was associated with a significantly higher risk of all-cause mortality and all-noncancer mortality, regardless of the duration of its use, and the magnitude of the hazard ratios was smaller with a longer duration of insulin use. On the other hand, the hazard ratios were all similar and not statistically significant for all-cancer mortality and all cancer other than breast cancer mortality. This was different from the disproportionately higher risk associated with insulin use for a longer duration of ≥3 years or ≥5 years for breast cancer mortality.

## 4. Discussion

The findings suggest that insulin use might be associated with a higher risk of breast cancer mortality if the duration of its use is ≥3 years (Table 3). Such an effect was consistently observed in the whole cohort analyses and the subgroup analyses and was specific for breast cancer mortality and not for cancers other than breast cancer (Table 3). Because there were only 5 cases of breast cancer mortality in patients with type 1 diabetes mellitus (Table 1 and Figure 1) and the results did not markedly change when only patients with type 2 diabetes mellitus were analyzed in secondary analyses (data not shown), it is believed that the results of the present study would best be applied to patients with type 2 diabetes mellitus.

In previous European studies, the link between insulin use (including human insulin and insulin glargine) and cancer (including breast cancer) risk was not conclusive [18–20]. As pointed out, the shortcomings of these studies included short follow-up duration, short duration of exposure to insulin, lack of evaluation of a dose-response relationship, lack of adjustment for major confounders such as body mass index, and use of secondary data. Most of these concerns (except for dose-response relationship) were addressed in the present study, which used a population-based follow-up design over a long duration with a large cohort of a nationally representative sample of patients with diabetes.

Strengths of the present study included a complete ascertainment of vital status by confirming data with the national death certificate database. The consistency in different analyses (Table 3) suggests a causal relationship. Furthermore, the temporal relationship between the cause (insulin exposure) and effect (breast cancer mortality) is true. The higher risk of breast cancer mortality seen in the patients with exposure to exogenous insulin for ≥3 or ≥5 years (Table 3) indicates an appropriate period for incubation, which should eliminate the concern of biological plausibility associated with a short duration of exposure.

Some might still argue that an exposure to insulin treatment for 3 or 5 years was too short for the development of breast cancer and its progression to mortality. In clinical practice insulin is always used subsequently and thereafter once it is prescribed. Therefore, the actual duration of insulin exposure in those who died of breast cancer in the present study should include the exposure period at baseline plus the follow-up duration before breast cancer mortality. Because the median duration of followup among those who died of breast cancer was 3.7 years, these patients should have been exposed to exogenous insulin for at least 6 years or 8 years, respectively, for those who had received insulin treatment for 3 or 5 years at baseline.

Another important issue regarding the plausibility is that since this study is essentially conducted in patients with type 2 diabetes mellitus, the hyperinsulinemia resulting from insulin resistance preceding the use of exogenous insulin might have exerted an adverse effect on the development of breast cancer. Therefore, the relatively short term effects of exogenous insulin could be explained by a preconditioning of long exposure to endogenous hyperinsulinemia as a result of insulin resistance since the prediabetes stage. Insulin receptors are expressed on breast epithelial cells and overexpressed on breast cancer cells [15]. Thus, the mitogenic effects of exogenous insulin can be exaggerated when insulin resistance and some precancerous or early cancerous lesions are present. When exogenous insulin was added, these lesions might progress rapidly to invasive cancer and lead to mortality from breast cancer after several years of its use. Such a speculation was supported by studies showing the development of more advanced-stage breast cancer [21] and a reduced early survival within the first 5 years following breast cancer [22] in women with diabetes.

In clinical practice, it may be difficult to deliver the exact dosage of insulin to meet physiological requirements. Therefore, patients receiving exogenous insulin in the presence of insulin resistance may be repetitively exposed to high levels of insulin, which may trigger the mitogenic pathways leading to cancer in susceptible patients. Endogenous hyperinsulinemia resulting from insulin resistance is predictive for the development of and mortality from breast cancer [10–14]. Studies also suggested a higher risk of breast cancer in women with prediabetes [23] and type 2 diabetes [24], both are characterized by endogenous hyperinsulinemia resulting from insulin resistance. This study adds to the literature by showing a link of exogenous insulin use in patients with diabetes to breast cancer mortality. Altogether, these observations suggested that hyperinsulinemia, whether of endogenous or exogenous origin, may promote the development and aggravate the progression of breast cancer.

It is worth mentioning that, at the time of enrollment of the diabetes cohort, insulin analogues were not yet available in Taiwan. No insulin analogues were in use in Taiwan before January 2004. Therefore, the findings of the present study on the link between insulin use (mainly human) and breast cancer mortality cannot be readily extrapolated to insulin analogues.

Obesity is a well-recognized risk factor for breast cancer [25, 26] and is also associated with a higher risk of mortality from breast cancer [27–29]. It is puzzling that the present study did not show a significant association between body mass index and breast cancer mortality. There are some possibilities that remain to be clarified. First, the present study recruited only patients with diabetes. Therefore the link between obesity and breast cancer mortality observed in the general population might not be readily extrapolated to patients with diabetes. Second, because most previous studies showing a link between obesity and breast cancer were conducted in western countries, it remains to be explored whether ethnic differences might exist. Third, it is common that obese individuals may self-report lower body weight but higher body height [30]. This might have led to a biased estimate of body mass index and its relationship to breast cancer mortality in the present study.

Diabetes control and obesity are known to be associated with more advanced or aggressive breast cancer [14, 31–33]. Insulin use often leads to increased body weight, and it is always used in a later stage of diabetes, when hyperglycemia cannot be adequately controlled by oral antidiabetic agents. Therefore, the use of insulin might be a marker for more severe diabetes or obesity, which could be the real factors affecting breast cancer mortality. In the present study, we did not have data on body weight or glycemic control status before and after the use of insulin for analyses of their impact. Future studies are required to clarify whether the breast cancer mortality related to insulin use can be ascribed to insulin* per se*.

The analyses on the relationship between different durations of insulin use and different causes of mortality (Table 3) might provide some important clues for a cause-effect relationship. For all-cause mortality and all-noncancer mortality, which can mostly be ascribed to macrovascular and microvascular complications of diabetes [34, 35], there was a significant association with insulin use, regardless of the duration of its use (Table 3). Additionally, the magnitudes of hazard ratios for these causes of mortality for a duration of insulin use of <3 (or <5) years were greater than those for ≥3 (or ≥5) years of insulin use (Table 3). This may not suggest a cause-effect relationship, taking into account the very short duration of insulin use in the lower exposure groups. Therefore, for these causes of mortality, insulin use may only be a surrogate for the real culprit of poor glycemic control, which has already been in existence for a long time before the initiation of insulin. Better control of hyperglycemia after a prolonged duration of insulin use may explain the attenuation of the hazard ratios for insulin use for ≥3 (or ≥5) years for all-cause mortality (mostly ascribed to diabetic vascular complications) and all-noncancer mortality (Table 3). On the other hand, a lack of association with all-cancer mortality and all cancer other than breast cancer mortality regardless of the duration of insulin use, together with an increase in magnitude in the hazard ratios for breast cancer mortality among users of insulin for a longer duration (for ≥3 [or ≥5] years) (Table 3), support a specific link and probably a causal relationship between insulin use and breast cancer mortality.

Study limitations included a lack of actual measurement of confounding factors such as age of menarche, menopause, use of hormone replacement therapy, concurrent use of metformin and other antidiabetic drugs, alcohol drinking, family history, lifestyle, dietary factors, and genetic parameters. Second, we did not have biochemical data such as hormonal profiles, blood glucose levels, hemoglobin A1C concentrations, insulin, C-peptide levels, or calculation of homeostasis model assessment for insulin resistance for evaluating their impact. Third, we were not able to evaluate the effects of insulin analogues (because of their late clinical use in Taiwan) and the different types of insulin such as regular insulin or neutral protamine Hagedorn (because of the lack of information). Fourth, we studied the link between insulin use and breast cancer mortality rather than its incidence. Because mortality and incidence are different clinical entities and may be linked to different factors, whether insulin use is a risk factor for breast cancer incidence awaits further investigation. Fifth, data related to the following were lacking: features of breast cancer such as the pathology, staging, grading, tumor size, and nodal involvement at diagnosis; estrogen receptor status and other molecular features; administered therapy; response to therapy; and related outcomes of local or distant recurrence. Finally, it should be pointed out that a clear dose-response relationship could not be demonstrated in the present study because of the lack of information on the amount of insulin use for these patients.

In summary, in a followup of a nationally representative cohort of female patients with diabetes, insulin use (mainly human insulin) for 3 years or more may be associated with breast cancer mortality, with a fully adjusted hazard ratio (95% confidence interval) of 2.006 (1.102–3.653). This study adds to the literature suggesting a link of insulin use to breast cancer mortality. Because of the limited numbers of breast cancer deaths and the lack of clear duration-risk relationship, future confirmation is required.

## Figures and Tables

**Table 1:** Baseline characteristics of women with diabetes who died and those who did not die of breast cancer.

Variable	Died of breast cancer		*P* value
	No	Yes	
*N* = 48,880	48,709	171	
Age, years	61.1 (11.6)	63.6 (9.9)	0.0013
Age, years			
<45	4,010 (8.2)	4 (2.3)	0.0050
45–64	25,251 (51.8)	84 (49.1)	
≥65	19,448 (39.9)	83 (48.5)	
Diabetes duration, years	7.5 (6.6)	8.5 (7.6)	0.0706
Diabetes type, % type 1	1,770 (3.6)	5 (2.9)	0.6204
Body mass index, kg/m^2^	24.7 (3.8)	24.3 (4.0)	0.2315
Smoking, % yes	1,669 (3.4)	6 (3.5)	0.9529
Use of insulin, %			
Non-users	43,546 (89.4)	150 (87.7)	0.1229
Use for <3 years	1,891 (3.9)	3 (1.8)	
Use for 3–4.9 years	842 (1.7)	5 (2.9)	
Use for ≥5 years	2,430 (5.0)	13 (7.6)	
Area of residence, % urban	22,374 (45.9)	94 (55.0)	0.0179
Median duration of follow-up, years	7.4	3.7	
Median duration of insulin use in insulin users, years	4.0	6.0	

Data are expressed as mean (SD) or *n* (%).

Median follow-up duration: 7.3 years and 7.5 years for insulin users and non-users, respectively.

**Table 2:** Cox proportional hazards models showing mutually adjusted hazard ratios and their 95% confidence intervals for breast cancer mortality in women with diabetes.

Variables	Interpretation	Mutually adjusted hazard ratio	95% confidence interval	*P* value
Age	Every 1-year increase	1.027	(1.012, 1.042)	0.0005
Diabetes duration	Every 1-year increase	1.017	(0.995, 1.040)	0.1298
Diabetes type	Type 2 vs. Type 1	1.418	(0.527, 3.814)	0.4887
Body mass index	Every 1-kg/m^2^ increase	0.973	(0.933, 1.015)	0.2091
Smoking	Yes vs. No	1.000	(0.442, 2.261)	0.9995
Insulin use	Yes vs. No	1.339	(0.782, 2.293)	0.2878
Area of residence	Urban vs. Rural	1.294	(0.955, 1.752)	0.0958

Sensitivity analyses^*∗*^				
Excluding patients who died of breast cancer within 3 years of diabetes onset				
Age	Every 1-year increase	1.031	(1.016, 1.047)	<0.0001
Diabetes duration	Every 1-year increase	1.020	(0.998, 1.043)	0.0719
Diabetes type	Type 2 vs. Type 1	1.378	(0.513, 3.706)	0.5250
Body mass index	Every 1-kg/m^2^ increase	0.976	(0.935, 1.020)	0.2780
Smoking	Yes vs. No	1.047	(0.462, 2.370)	0.9126
Insulin use	Yes vs. No	1.384	(0.806, 2.375)	0.2387
Area of residence	Urban vs. Rural	1.333	(0.976, 1.819)	0.0705

^*∗*^Sensitivity analyses conducted after excluding patients who died of breast cancer within 5 years and 7 years of diabetes onset separately did not remarkably change the adjusted hazard ratios.

**Table 3:** Multivariable-adjusted hazard ratios and their 95% confidence intervals for various causes of mortality with regard to cutoffs of duration of insulin use at 3 years and 5 years.

Cohort/Dependent variable	Duration of insulin use					
	HR	95% CI	*P* value	HR	95% CI	*P* value
		<3 years			≥3 years	

(i) Whole cohort analyses^#^						
Breast cancer mortality^*∗*^	0.567	(0.179, 1.791)	0.3333	2.006	(1.102, 3.653)	0.0228
Breast cancer mortality^*∗∗*^				2.085	(1.153, 3.769)	0.0150
All-cause mortality^*∗*^	1.696	(1.573, 1.829)	<0.0001	1.453	(1.353, 1.559)	<0.0001
All-noncancer mortality^*∗*^	1.811	(1.672, 1.962)	<0.0001	1.509	(1.400, 1.627)	<0.0001
All-cancer mortality^*∗*^	1.076	(0.847, 1.367)	0.5479	1.123	(0.910, 1.386)	0.2793
All cancer other than breast cancer mortality^*∗*^	1.118	(0.875, 1.427)	0.3728	1.049	(0.838, 1.313)	0.6776
(ii) Subgroup analyses^##^						
Breast cancer mortality^*∗*^	0.473	(0.151, 1.487)	0.2004	1.898	(1.097, 3.282)	0.0219
Breast cancer mortality^*∗∗*^				1.957	(1.138, 3.366)	0.0152

	<5 years			≥5 years		

(i) Whole cohort analyses^#^						
Breast cancer mortality^*∗*^	1.045	(0.505, 2.162)	0.9048	1.899	(0.934, 3.860)	0.0763
Breast cancer mortality^*∗∗*^				1.885	(0.936, 3.799)	0.0761
All-cause mortality^*∗*^	1.702	(1.596, 1.815)	<0.0001	1.334	(1.229, 1.449)	<0.0001
All-noncancer mortality^*∗*^	1.821	(1.701, 1.949)	<0.0001	1.620	(1.247, 1.487)	<0.0001
All-cancer mortality^*∗*^	1.059	(0.862, 1.300)	0.5864	1.170	(0.922, 1.485)	0.1957
All cancer other than breast cancer mortality^*∗*^	1.058	(0.854, 1.311)	0.6044	1.109	(0.862, 1.429)	0.4206
(ii) Subgroup analyses^##^						
Breast cancer mortality^*∗*^	0.916	(0.448, 1.871)	0.8088	1.818	(0.941, 3.512)	0.0751
Breast cancer mortality^*∗∗*^				1.832	(0.952, 3.524)	0.0699

HR: hazard ratio, CI: confidence interval.

^#^Whole-cohort analyses were conducted in 48,880 female patients with diabetes; models were adjusted for age, diabetes type, diabetes duration, body mass index, smoking, and area of residence.

^##^Subgroup analyses were conducted in 171 patients with breast cancer mortality and 855 randomly selected patients without breast cancer mortality matched for diabetes duration; models are adjusted for age, diabetes type, body mass index, smoking, and area of residence.

^*∗*^Referent group: women with diabetes not using insulin.

^*∗∗*^Referent group: women with diabetes not using insulin or using insulin for <3 (or <5) years.
